# Supplementary material for: The Role of the Mind-Body Connection in Children with Food Reactions and Identified Adversity: Implications for Integrating Stress Management and Resilience Strategies in Clinical Practice
Source: Children (Basel). 2023 Mar 16;10(3):563. doi: 10.3390/children10030563 (PMC10047561; doi:10.3390/children10030563)
Supplement: Supplementary file 1 [file children-10-00563-s001.zip › children-2215723-supplementary.pdf]

Supplementary Figure S1: Number of Patients Using Integrative Modalities

Supplementary Figure S1 displays the integrative modality used by patients upon intake on the x-axis and the number of patients on the y-axis. The numbers reflected are inclusive of patients who used more than one integrative modality. The most commonly used integrative modality upon intake was nutrition (28%, n=69). Other integrative modalities included physical therapy or occupational therapy, aromatherapy, chiropractic, music, holistic or naturopathic physician, prayer, biofeedback, mental health services, yoga, meditation, homeopathy, osteopathy, acupuncture, guided imagery, breathing, distraction, and massage. Mental health services included visits with a psychologist, psychiatrist, or special type of therapy (e.g. cognitive behavioral therapy).

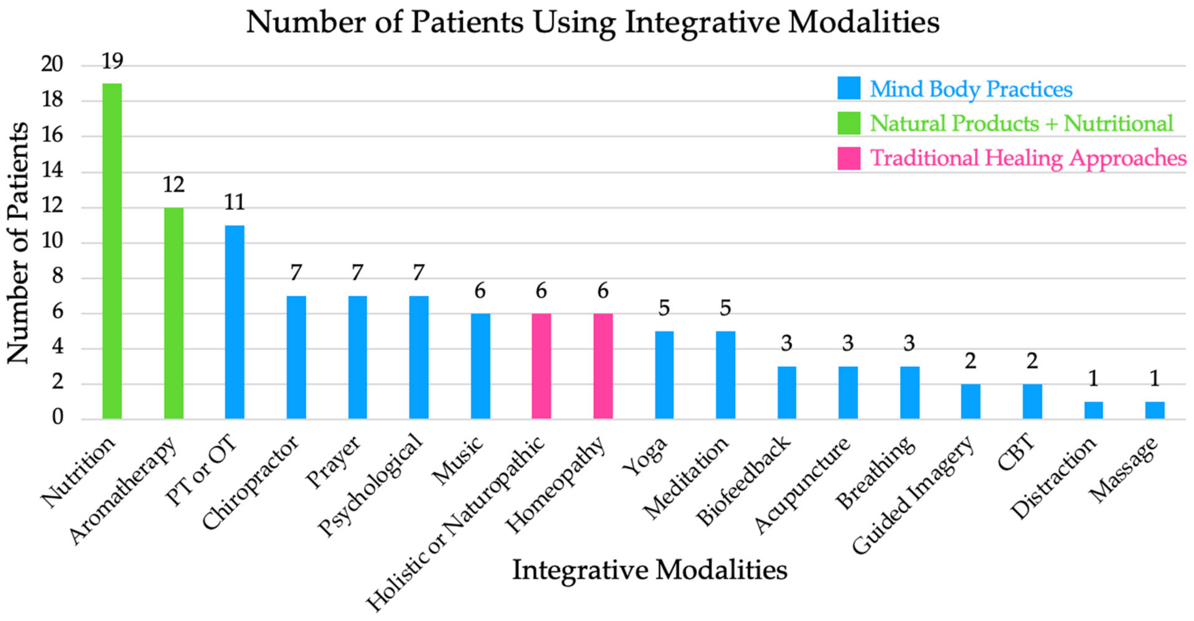

Supplementary Table S1. Correlation between Presence of Stressors and Usage of Integrative Modalities without Physical Therapy

	**Stressors - Y (n=98)**	**Stressors - N (n=32)**
**Integrative Methods (no PT) - Y**	56 (57%)	10 (31%)
**Integrative Methods (no PT) - N**	42 (43%)	22 (69%)
